# Rapid Restoration of Circulating Vitamin B12 Levels by Using Oral Sucrosomial^®^ Vitamin B12 in Metformin-Associated B12 Deficiency: Results from a Double-Blind, Placebo-Controlled Randomized Clinical Trial

**DOI:** 10.3390/pharmaceutics18020237

**Published:** 2026-02-13

**Authors:** Gabriele Conti, Allah W. Kalo, Ikram Ujjan, Aisha Aslam, Amjad Khan, Nazia M. Memon, Shair Z. Kakar, Naseer Ahmed, Shifa Zahid, Germano Tarantino, Elisa Brilli

**Affiliations:** 1 Human Microbiomics Unit, Department of Medical and Surgical Sciences, University of Bologna, 40138 Bologna, Italy; gabriele.conti12@unibo.it; 2 Department of Community Medicine, Bilawal Medical College, Liaquat University of Medical and Health Sciences (LUMHS), Jamshoro 76090, Pakistan; 3 Department of Pathology, Liaquat University of Medical and Health Sciences (LUMHS), Jamshoro 76090, Pakistan; ayeshaaslam62@gmail.com (A.A.); naziamumtaz@lumhs.edu.pk (N.M.M.); shifabhatti373@gmail.com (S.Z.); 4 Department of Biochemistry, Liaquat University of Medical and Health Sciences (LUMHS), Jamshoro 76090, Pakistan; 5 Nuffield Division of Clinical Laboratory Sciences (NDCLS), Radcliffe Department of Medicine, University of Oxford, Oxford OX1 2JD, UK; 6 Endocrine Department, Bolan Medical Complex Hospital, Quetta 87300, Pakistan; 7 Department of Gastroenterology, Aria Institute of Medical Sciences, Quetta 87300, Pakistan; nasierahmedkhan@gmail.com; 8 R&D Department, PharmaNutra S.p.A., 56122 Pisa, Italy

**Keywords:** vitamin B12 deficiency, type 2 diabetes mellitus, metformin, oral vitamin B12 delivery system, Sucrosomial^®^ vitamin B12

## Abstract

**Background/Objectives:** Vitamin B12 deficiency is common in individuals with type 2 diabetes mellitus (T2DM) receiving long-term metformin therapy, primarily due to impaired intestinal absorption. Conventional oral B12 supplementation is often associated with delayed or inconsistent biochemical correction. A lipid-based Sucrosomial^®^ delivery system has been shown to improve circulatory vitamin B12 levels in healthy adults with deficiency, and the present study evaluates its performance in the clinically challenging context of metformin-treated individuals with T2DM, a population characterized by pharmacologically impaired intestinal vitamin B12 absorption. **Methods:** This multicentre, double-blind, placebo-controlled, parallel-group randomized clinical trial evaluated the efficacy, safety, and tolerability of a Sucrosomial^®^ vitamin B12 formulation in adults with T2DM receiving metformin and presenting with vitamin B12 deficiency. Participants were randomized (1:1) to receive oral Sucrosomial^®^ vitamin B12 (1000 µg daily; *n* = 25) or placebo (*n* = 25) for three weeks. Serum total vitamin B12 and holotranscobalamin (HoloTC), the biologically active fraction of vitamin B12, were assessed at baseline and during follow-up, with time-to-normalization and safety analyses performed. **Results:** Sucrosomial^®^ vitamin B12 supplementation resulted in rapid and sustained increases in circulating vitamin B12 levels, with early separation from placebo, and a substantially higher proportion of participants achieved normalization of serum vitamin B12 concentrations within the three-week period. Parallel improvements in HoloTC levels indicated enhanced biologically active vitamin B12 availability, and the intervention was well tolerated with no clinically relevant safety concerns. **Conclusions:** These findings demonstrate that an oral Sucrosomial^®^ vitamin B12 formulation can achieve rapid and reliable biochemical repletion of both total and biologically active vitamin B12 in metformin-treated adults with T2DM, despite pharmacologically impaired intestinal absorption, while maintaining a favourable safety and tolerability profile.

## 1. Introduction

Vitamin B12 (cobalamin) is an essential water-soluble vitamin required for DNA synthesis, erythropoiesis, and neurological function [1,2,3]. Deficiency can lead to megaloblastic anemia, peripheral neuropathy, cognitive impairment, and a range of metabolic disturbances [3,4,5]. Despite its fundamental biological role, vitamin B12 deficiency remains prevalent worldwide, particularly among older adults, individuals with gastrointestinal disorders, and those following restrictive dietary patterns [6,7,8,9,10]. Importantly, vitamin B12 deficiency is also frequently observed in people living with Type 2 Diabetes Mellitus (T2DM), especially among individuals receiving long-term metformin therapy [11,12,13,14,15,16,17].

Under physiological conditions, dietary vitamin B12 absorption is a complex, tightly regulated multistep process. Vitamin B12 is released from food proteins in the acidic environment of the stomach, where it initially binds to salivary haptocorrins (R-proteins). Following pancreatic proteolysis of haptocorrins in the duodenum, vitamin B12 is transferred to intrinsic factor (IF), forming a stable vitamin B12–IF complex. This complex is subsequently absorbed in the terminal ileum via receptor-mediated endocytosis involving the cubilin–amnionless receptor complex expressed on enterocytes. After cellular uptake, vitamin B12 enters the circulation predominantly bound to transcobalamin II, which delivers the biologically active fraction of the vitamin to peripheral tissues. This transcobalamin-bound fraction, referred to as holotranscobalamin (HoloTC), represents the metabolically active form of vitamin B12 available for cellular uptake and is considered a sensitive indicator of functional vitamin B12 status. Disruption at any stage of this tightly coordinated pathway—whether due to pharmacological interference, gastrointestinal pathology, or altered intestinal physiology—can substantially impair vitamin B12 bioavailability and systemic availability [3,7,10,18].

Metformin is a first-line oral hypoglycaemic agent for T2DM and is prescribed to an estimated 150 million individuals globally [19,20]. However, chronic metformin use is strongly associated with vitamin B12 deficiency [11,12,13,14,15,16,17]. The pathogenesis of metformin-associated vitamin B12 deficiency is multifactorial and is primarily attributed to impaired intestinal absorption in the distal ileum [13,21]. The predominant mechanism involves interference with the calcium-dependent binding of the vitamin B12–intrinsic factor complex to the cubilin receptor, a critical step for cellular uptake of the vitamin [21,22]. Additional contributory mechanisms include metformin-related alterations in gastrointestinal motility that may predispose to small intestinal bacterial overgrowth, thereby increasing bacterial competition for luminal vitamin B12 [13,23]. Less consistently supported mechanisms include reduced IF secretion by gastric parietal cells and altered hepatic vitamin B12 handling [24]. Epidemiological studies suggest that 10–30% of individuals receiving metformin develop biochemical vitamin B12 deficiency, with an even greater proportion exhibiting sub-optimal concentrations [13,25,26]. This is clinically relevant, as vitamin B12 deficiency may contribute to or exacerbate diabetic peripheral neuropathy, complicating clinical assessment and potentially worsening neurological outcomes [13,23,27,28].

Oral vitamin B12 supplementation is widely used for the management of vitamin B12 deficiency [5,10,29,30,31]. However, conventional oral formulations are associated with variable and often unpredictable absorption, largely due to their reliance on intact IF–mediated uptake, susceptibility to degradation in the gastric environment, and constraints imposed by gastrointestinal physiology [18,31]. These limitations are particularly relevant in individuals receiving long-term metformin therapy, in whom IF–dependent absorption is compromised.

Sucrosomial^®^ vitamin B12 represents an alternative vitamin B12 based supplement developed to enhance cobalamin stability during gastrointestinal transit and to support more consistent absorption [32]. Sucrosomial^®^ technology, allows vitamin B12 to be incorporated into a structured named “sucrosome”that is composed of a phospholipid matrix, primarily derived from sunflower lecithin, combined with sucrose ester (sucrester) and tricalcium phosphate, forming a protective system. This architecture is intended to shield vitamin B12 from degradation during gastric transit and to facilitate its interaction with the intestinal epithelium, thereby improving intestinal absorption of the micronutrient. By reducing reliance on calcium-dependent IF–mediated interactions at the intestinal epithelium, including those involving the cubilin receptor complex, the Sucrosomial^®^ technology helps mitigate absorption limitations associated with pharmacological interference. From a nutritional delivery perspective, the Sucrosomial^®^ technology system represents a food-grade like delivery system to enhance the absorption of bioactive micronutrients while potentially reducing dosing requirements and gastrointestinal burden.

Consistent with this rationale, a previous multicentre, double-blind randomized clinical trial in healthy adults with deficient or borderline vitamin B12 levels demonstrated that Sucrosomial^®^ vitamin B12 induced rapid and marked increases in circulatory vitamin B12 concentrations within 24 h, with sustained elevations over one week, compared with several conventional oral formulations [32]. Although the precise intestinal absorption pathways of Sucrosomial^®^ vitamin B12 in humans have not been fully elucidated, the Sucrosomial^®^ technology has been shown to improve systemic exposure of several micronutrients, including iron, magnesium, and vitamin D3, in both preclinical and clinical settings [33,34,35,36,37]. Preclinical and ex vivo studies indicate that the “sucrosome” structure retains its integrity during simulated gastrointestinal conditions and can interact with the intestinal epithelium via paracellular and transcellular transport mechanisms, including uptake by enterocytes and M cells [35,36,37,38]. These findings support the biological plausibility of sucrosomial^®^ delivery as a strategy to enhance oral micronutrient uptake, particularly in settings where conventional absorption may be compromised.

Existing studies investigating vitamin B12 supplementation strategies in metformin-treated individuals with T2DM are limited in number and are constrained by important methodological and conceptual limitations [27,39,40,41,42,43,44]. Extended-duration trials of oral vitamin B12 supplementation have primarily focused on long-term neurological outcomes in highly selected patient populations, providing limited insight into the early correction of vitamin B12 deficiency and typically requiring prolonged treatment durations or high cumulative exposure. Other investigations have relied on alternative routes of administration, including intramuscular, sublingual, or intranasal delivery, achieving biochemical correction by bypassing gastrointestinal absorption altogether and therefore not addressing whether the intestinal malabsorption central to metformin-associated vitamin B12 deficiency can be overcome through oral delivery. Moreover, most of these studies were open-label, lacked placebo control, or did not systematically evaluate early response kinetics or the proportion of patients achieving normalization over short treatment periods. Collectively, these limitations leave unresolved whether an optimized oral vitamin B12 delivery strategy can rapidly, reliably, and safely restore circulating vitamin B12 levels in metformin-treated T2DM patients.

Building on prior clinical evidence demonstrating rapid circulatory vitamin B12 repletion with an oral Sucrosomial^®^ vitamin B12 in healthy deficient adults [32], the present study was designed to determine whether comparable efficacy could be achieved in a clinically more challenging population characterized by metformin-associated malabsorption. Specifically, the trial evaluated whether Sucrosomial^®^ vitamin B12 could achieve rapid and consistent restoration of circulating vitamin B12 levels in adults with T2DM receiving metformin therapy, in whom vitamin B12 absorption is pharmacologically compromised, while also assessing safety and tolerability under controlled conditions.

## 2. Materials and Methods

### 2.1. Study Design and Setting

This study was conducted as a prospective, multi-centre, double-blind, placebo-controlled, parallel-group, randomized clinical trial at Liaquat University of Medical and Health Sciences (LUMHS), Jamshoro, and Bolan Medical Complex, Hospital, Quetta, Pakistan. The trial was carried out from 11 August 2025 to 3 December 2025, following the principles of the Declaration of Helsinki, Good Clinical Practice (GCP) guidelines, and institutional ethical standards. Ethical approval was granted by the Research Ethics Committee of LUMHS (No. LUMHS/REC//733, dated 9 May 2025). The trial was prospectively registered at ClinicalTrials.gov (Identifier: NCT06983223). Written informed consent was obtained from all participants prior to enrolment.

### 2.2. Participants

Inclusion Criteria

Eligible participants were adults aged 18 to 75 years with a confirmed diagnosis of T2DM who had been receiving metformin therapy for at least one year. Participants were required to have deficient or sub-optimal serum vitamin B12 levels (150–320 pg/mL) at screening and not be taking vitamin B12 supplements at screening or within the preceding three weeks.

Exclusion Criteria

Participants were excluded if they had a known diagnosis of pernicious anemia or other medical conditions associated with impaired vitamin B12 absorption, such as Crohn’s disease or celiac disease. Individuals with a history of gastric surgery or small intestine resection, or those receiving medications known to interfere with vitamin B12 absorption (other than metformin), were also excluded. Additional exclusion criteria included severe renal impairment (eGFR < 30 mL/min/1.73 m^2^), end-stage renal disease, severe hepatic dysfunction (ALT or AST more than three times the upper limit of normal), pregnancy or breastfeeding, or documented hypersensitivity to vitamin B12 treatment. Participants who had taken part in another clinical trial within the preceding three weeks were also excluded.

### 2.3. Randomization and Blinding

Participants were randomized in a 1:1 ratio to receive either Sucrosomial^®^ vitamin B12 or a placebo using a computer-generated allocation sequence. Allocation was concealed through sequentially numbered sachets prepared by an independent pharmacist not involved in study procedures. The active product and placebo were indistinguishable in appearance, taste, and packaging to ensure blinding. Participants, healthcare providers, investigators, and outcome assessors remained blinded to treatment assignment until completion of data analysis.

### 2.4. Intervention

Participants assigned to the intervention group received Sucrosomial^®^ Vitamin B12 (1000 µg PharmaNutra S.p.A., Pisa, Italy) in an oro-dissolvable sachet, administered once daily for three weeks. The control group received a matching placebo sachet identical in appearance, taste, and packaging. Participants were instructed to place the sachet directly on the tongue and allow it to dissolve; a small sip of water could be taken if needed to aid dissolution. A conventional oral vitamin B12 comparator arm was not included, as the study was designed as a placebo-controlled proof-of-efficacy trial to isolate the biochemical response attributable to the Sucrosomial^®^ delivery strategy in metformin-treated individuals; comparative efficacy versus conventional oral vitamin B12 formulations has been previously established in vitamin B12–deficient adults [32].

All participants continued their usual metformin therapy and routine diabetes care throughout the study period. They were advised to maintain their habitual diet and avoid initiating any new vitamin B12–fortified foods or dietary supplements during the trial.

If a participant’s serum vitamin B12 concentration reached or exceeded the upper limit of the normal laboratory reference range at any follow-up timepoint (24 h, week 1, or week 2), further supplementation was discontinued for that individual as a safety measure.

If a participant’s serum vitamin B12 concentration reached or exceeded 700 pg/mL, a predefined pragmatic and conservative upper-normal target within the accredited laboratory reference range (234–894 pg/mL, although reference ranges may vary slightly between laboratories), at any follow-up timepoint (24 h, week 1, or week 2), further supplementation was discontinued for that individual as a protocol-defined safety and adequacy measure. This threshold was selected to indicate sufficient biochemical repletion while avoiding unnecessary continued high-dose supplementation, particularly in a population with pharmacologically impaired intestinal absorption. Published clinical practice recommendations indicate that serum vitamin B12 concentrations above approximately 300–400 pg/mL are generally considered adequate [45,46,47,48], while higher upper-normal targets are often used in high-risk populations to ensure biochemical repletion. Importantly, this protocol-defined discontinuation criterion was implemented for study standardization and participant safety and does not imply a requirement for routine intensive monitoring or dose adjustment in routine clinical practice once deficiency has been corrected. The use of 700 pg/mL to confirm adequacy of vitamin B12 status is consistent with clinical practice in high-risk populations and prior interventional studies evaluating oral vitamin B12 repletion strategies [27,40,41,44,45,46,47,48].

Adherence was assessed by sachet counts and participant self-report at each follow-up visit. To support adherence and monitor safety, study staff placed weekly reminder phone calls to participants during the intervention period; these calls reinforced dosing instructions, captured interim adverse events, and answered participant queries.

### 2.5. Laboratory Procedures

Venous blood samples were collected at baseline, 24 h, week 1, week 2, and week 3. Samples were processed according to standard laboratory protocols, with serum separated by centrifugation and analyzed in the LUMHS Central Diagnostic and Research Laboratory, Hyderabad, Pakistan. Serum vitamin B12 concentrations were measured using a validated chemiluminescent immunoassay, following the manufacturer’s specifications. Serum HoloTC (active vitamin B12) concentrations were determined using the Elecsys^®^ Active B12 electrochemiluminescence immunoassay (ECLIA) (Roche Diagnostics, Basel, Switzerland), performed on cobas e immunoassay analyzers, according to the manufacturer’s instructions. Internal and external quality control procedures were implemented to ensure accuracy and precision across all timepoints. All laboratory personnel were blinded to treatment allocation to prevent measurement bias.

### 2.6. Sample Size

A total of 50 participants were enrolled in this trial. The sample size was determined using serum vitamin B12 changes previously observed in our multicentre randomized trial of Sucrosomial vitamin B12, where daily 1000 µg supplementation led to increases of 200–300 pg/mL over one week [32]. In the absence of prior placebo-controlled effect size estimates specific to metformin-treated T2DM populations, these data were considered an appropriate reference for powering a study focused on biochemical correction of vitamin B12 deficiency. On this basis, a sample size of 50 participants (25 per group) was considered adequate to detect clinically meaningful between-group differences in serum vitamin B12 concentrations across the 3-week intervention in correcting metformin-associated vitamin B12 deficiency, while accounting for the distinct absorptive challenges associated with metformin therapy. Importantly, the effect sizes observed in the present trial now provide population-specific estimates that can inform future prospectively powered randomized studies tailored to metformin-treated T2DM cohorts.

### 2.7. Outcome Measures

The study’s primary outcome was the change in circulatory vitamin B12 concentration from baseline to week 3, as well as the proportion of participants achieving normalization (upper-normal laboratory reference range) of serum vitamin B12 levels by week 3.

Secondary outcomes included the incidence of adverse events, along with treatment adherence and overall tolerability throughout the study period.

Exploratory analyses assessed the association between changes in total serum vitamin B12 and HoloTC concentrations over time to support the interpretation of biochemical response.

### 2.8. Statistical Analysis

All statistical analyses and graphical visualizations were performed using R software (version 4.5.0; R Foundation for Statistical Computing, Vienna, Austria), using the packages *microbAIDeR*, *ggplot2*, *lme4*, *lmerTest*, *emmeans*, *survival*, and *survminer* [49,50,51,52,53,54,55]. Continuous variables were assessed for normality using the Shapiro–Wilk test and visual inspection of distribution plots. Data are reported as mean ± standard error of the mean (SEM) for approximately normally distributed variables, or as median ± standard error of the median (SEMed) for non-normally distributed variables, while categorical variables are presented as counts and percentages. Baseline characteristics were compared between treatment groups using independent samples *t*-tests or Wilcoxon rank-sum tests for continuous variables, as appropriate, and Fisher’s exact test for categorical variables.

Longitudinal changes in serum concentrations of vitamin B12 and its active form, HoloTC, were analyzed using linear mixed-effects models (LMM) with log-transformed biomarker values as the dependent variable. Fixed effects included study group, timepoint, and their interaction, with age, sex, and body mass index (BMI) included as covariates; a random intercept for participant ID was used to account for within-subject correlation. Type III analysis of variance was applied to test main effects and interactions. Discontinuation of supplementation upon reaching the clinical threshold was protocol-defined and addressed in the statistical analysis using linear mixed-effects models, which incorporate all available repeated measurements and are robust to unbalanced longitudinal data. Post hoc pairwise comparisons of estimated marginal means (emmeans) were conducted using false discovery rate (FDR)–adjusted *p*-values.

The timing of achievement of adequate to upper-normal laboratory reference range serum vitamin B12 concentrations was evaluated using cumulative incidence analysis. The event was defined as the first study visit at which serum vitamin B12 concentrations reached or exceeded the predefined adequate/upper-normal laboratory reference range (700 pg/mL). Participants who discontinued supplementation after achieving the predefined biochemical target continued scheduled follow-up assessments, and their last observed values were retained in all longitudinal analyses. Those who did not achieve the threshold during the 3-week follow-up were censored at the final study visit. Between-group differences in time to achievement were assessed using Cox proportional hazards regression, and cumulative incidence curves were generated for graphical representation.

All statistical tests were two-sided, and statistical significance was set at *p* ≤ 0.05, with trends reported at *p* ≤ 0.1. Significance levels were indicated in figures using the conventional star system (*p* ≤ 0.1, °; *p* ≤ 0.05, *; *p* ≤ 0.01, **; *p* ≤ 0.001, ***; *p* ≤ 0.0001, ****).

## 3. Results

**Figure 1:**
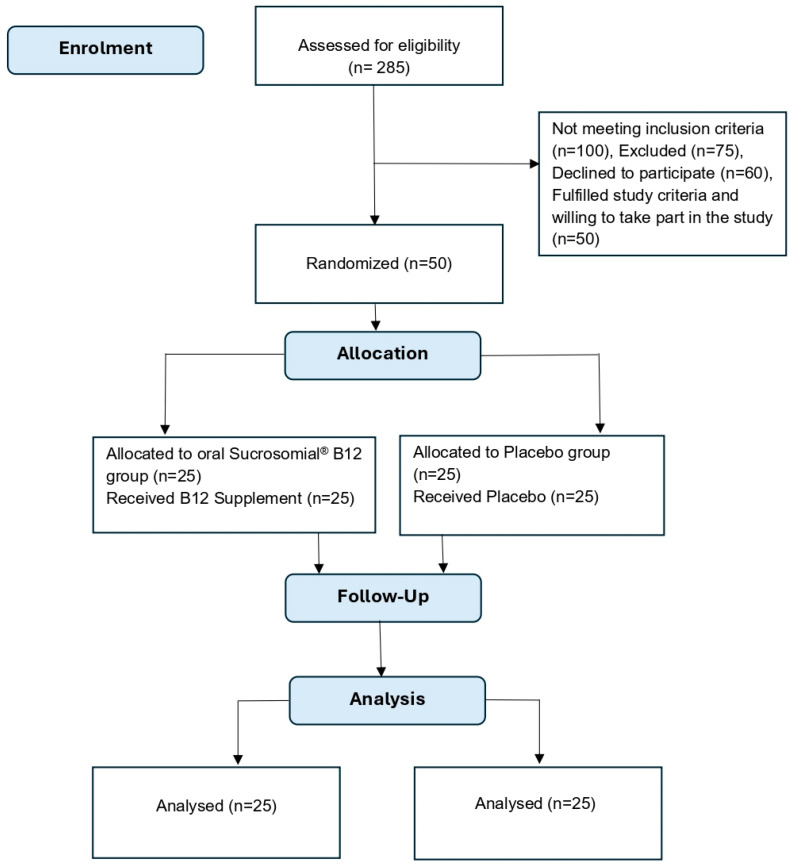
Study CONSORT flow diagram.

Participant flow through the study is summarized in the CONSORT flow diagram (Figure 1).

Baseline demographic and clinical characteristics of the study population are summarized in Table 1. The two study groups were comparable across most demographic, anthropometric, lifestyle, and medical history variables. Participants allocated to the Sucrosomial^®^ vitamin B12 group were older and had a longer duration of T2DM compared with the placebo group. A higher prevalence of diabetic neuropathy was observed in the placebo group. No other significant differences were detected between groups. Importantly, baseline total and active vitamin B12 concentrations were similar between groups.

### 3.1. Rapid and Sustained Increases in Circulatory Vitamin B12 Levels with Sucrosomial^®^ Vitamin B12 Compared with Placebo

Type III analysis of variance from the linear mixed-effects model showed a strong main effect of the studied formulations (F = 60.59, *p* < 0.0001) and timepoint (F = 26.06, *p* < 0.0001) on log-transformed serum vitamin B12 concentrations, together with a significant studied formulations × timepoint interaction (F = 12.48, *p* < 0.0001), indicating distinct temporal trajectories between treatment groups. Among covariates, age category showed a modest association with vitamin B12 levels (F = 4.36, *p* = 0.03), whereas sex and BMI category were not significantly associated, indicating that differences in vitamin B12 concentrations were primarily driven by treatment and time rather than confounding factors.

**Figure 2:**
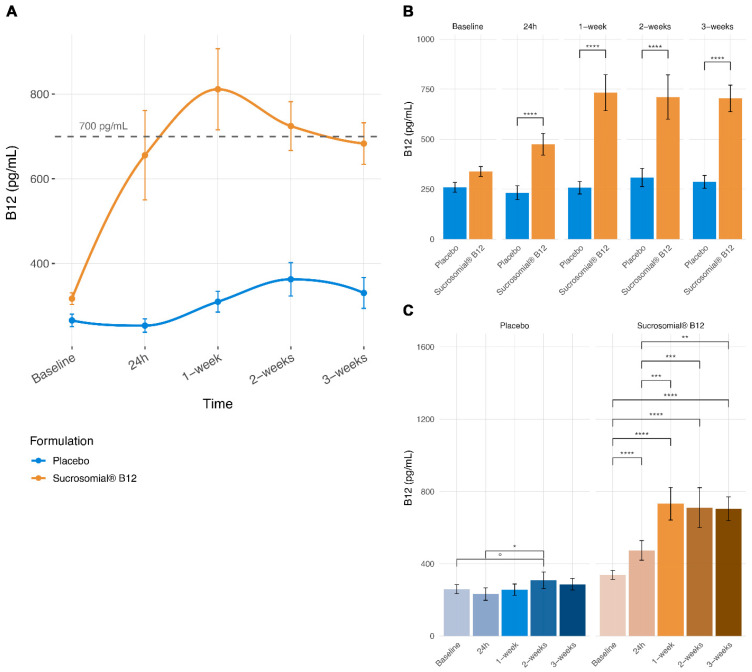
Longitudinal and comparative analyses of serum vitamin B12 concentrations. (**A**) Connected scatterplots showing mean serum vitamin B12 concentrations (±SEM) over time in the Sucrosomial^®^ Vitamin B12 and placebo groups. Lines illustrate group-level temporal trends. Individual participants exceeding predefined adequacy thresholds (e.g., ≥700 pg/mL) may not be visually apparent in group mean plots. (**B**) Barplots showing between-group differences in serum vitamin B12 concentrations at each timepoint. (**C**) Barplots showing within-group differences across timepoints for each formulation. Statistical comparisons were performed using LMM, and *p*-values were adjusted for multiple testing using the FDR method. At follow-up visits, values reflect only participants who had not yet reached the predefined clinical threshold (serum vitamin B12 ≥ 700 pg/mL) at earlier timepoints and therefore continued supplementation, as per study protocol. Significance levels are indicated as follows: *p* ≤ 0.1, °; *p* ≤ 0.05, *; *p* ≤ 0.01, **; *p* ≤ 0.001, ***; *p* ≤ 0.0001, ****. Numbers of participants contributing data at each timepoint were as follows: Placebo group Baseline *n* = 25, 24 h *n* = 25, 1-week *n* = 25, 2-week *n* = 25, 3-week *n* = 22; Sucrosomial^®^ vitamin B12 group Baseline *n* = 25, 24 h *n* = 25, 1-week *n* = 21, 2-week *n* = 10, 3-week *n* = 5.

Consistent with these findings, post hoc comparisons (Figure 2A,B) revealed no significant difference between groups at baseline, whereas serum vitamin B12 concentrations were significantly higher in the Sucrosomial^®^ Vitamin B12 group compared with placebo as early as 24 h after treatment initiation, corresponding to a 115% greater ratio of change (ROC) versus placebo. This relative difference peaked at 1 week (ROC: 117%, FDR-adjusted *p* < 0.0001) and was lower at later timepoints (ROC: 64% at 2 weeks and 70% at 3 weeks, all FDR-adjusted *p* < 0.0001), reflecting the protocol-mandated discontinuation of supplementation in participants who achieved upper-normal serum vitamin B12 concentrations and the resulting reduction in the number of evaluable subjects within the Sucrosomial^®^ group.

Within-group analyses (Figure 2A,C) in the placebo group showed modest and delayed increases in serum vitamin B12 concentrations at later follow-up visits, with a maximum relative increase of approximately 38.5% observed at 2 weeks. However, statistically significant differences were detected only for the comparison between 24 h and 2 weeks (FDR-adjusted *p* = 0.019), while the remaining comparisons did not reach statistical significance, indicating the absence of a consistent or progressive temporal trend. In contrast, participants receiving Sucrosomial^®^ Vitamin B12 demonstrated a rapid and pronounced increase from baseline already at 24 h (+105%), with further significant increases at 1 week (+156%), followed by persistently elevated levels at 2 weeks (+127%) and 3 weeks (+114%) relative to baseline (all FDR-adjusted *p* < 0.001). No significant differences were observed among later follow-up timepoints within the Sucrosomial^®^ group, indicating early attainment of elevated vitamin B12 concentrations followed by stabilization.

### 3.2. Rapid Cumulative Incidence of Achieving Upper-Normal Laboratory Reference Range Circulatory Vitamin B12 Levels and Cox Regression Analysis

**Figure 3:**
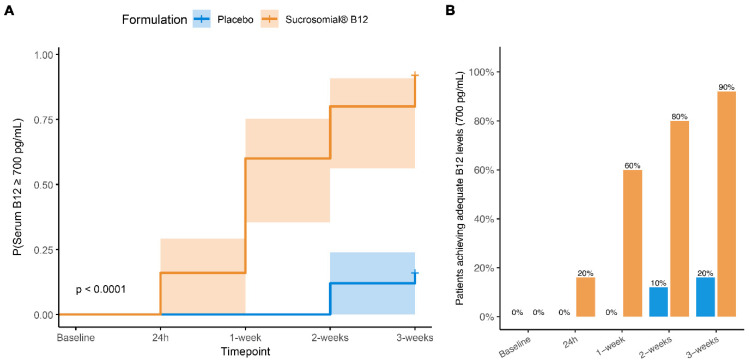
Cumulative incidence of achieving adequate serum vitamin B12 concentration levels. (**A**) Cumulative incidence curves showing the probability of achieving upper-normal laboratory reference range circulatory vitamin B12 concentrations over time in participants receiving Sucrosomial^®^ Vitamin B12 and placebo. This analysis reflects individual-level events and time-to-threshold attainment. The event was defined as the first study visit at which circulatory vitamin B12 concentrations reached or exceeded the predefined adequate/upper-normal laboratory reference range threshold (700 pg/mL). Between-group differences were assessed using Cox proportional hazards regression. (**B**) Barplots showing the cumulative percentage of participants reaching the adequate/upper-normal laboratory reference range of circulatory B12 at each follow-up timepoint.

The timing of achievement of upper-normal circulatory vitamin B12 concentrations differed markedly between treatment groups. Cumulative incidence analysis (Figure 3A) showed a substantially higher and earlier probability of reaching upper-normal laboratory reference range vitamin B12 levels in participants receiving Sucrosomial^®^ Vitamin B12 compared with placebo. By 1 week, 60% of participants in the Sucrosomial^®^ group had achieved upper-normal laboratory reference range vitamin B12 concentrations, increasing to 80% at 2 weeks and 90% at 3 weeks, whereas the corresponding proportions in the placebo group remained low throughout follow-up (10% at 2 weeks and 20% at 3 weeks; Figure 3B).

Consistently, Cox proportional hazards regression demonstrated a significantly greater likelihood of achieving upper-normal laboratory reference range circulatory vitamin B12 concentrations in the Sucrosomial^®^ Vitamin B12 group compared with placebo (hazard ratio [HR] = 14.19, 95% CI: 4.79–42.04; *p* < 0.0001). The model showed good discriminatory performance (concordance = 0.8), and global likelihood ratio, Wald, and log-rank tests all confirmed a robust between-group difference (all *p* < 0.0001).

### 3.3. Rapid and Sustained Increases in Circulatory Holotranscobalamin (HoloTC) Levels (Active Vitamin B12) with Sucrosomial^®^ Vitamin B12 Compared with Placebo

Type III analysis of variance from the linear mixed-effects model showed a significant main effect of study group (F = 26.74, FDR-adjusted *p* < 0.0001) and timepoint (F = 9.57, FDR-adjusted *p* < 0.0001) on log-transformed circulatory HoloTC concentrations, together with a significant study group × timepoint interaction (F = 12.50, FDR-adjusted *p* < 0.0001), indicating distinct temporal trajectories between treatment groups. No significant associations were observed for age category, sex, or BMI category, suggesting that differences in HoloTC concentrations were primarily driven by treatment and time rather than confounding factors.

**Figure 4:**
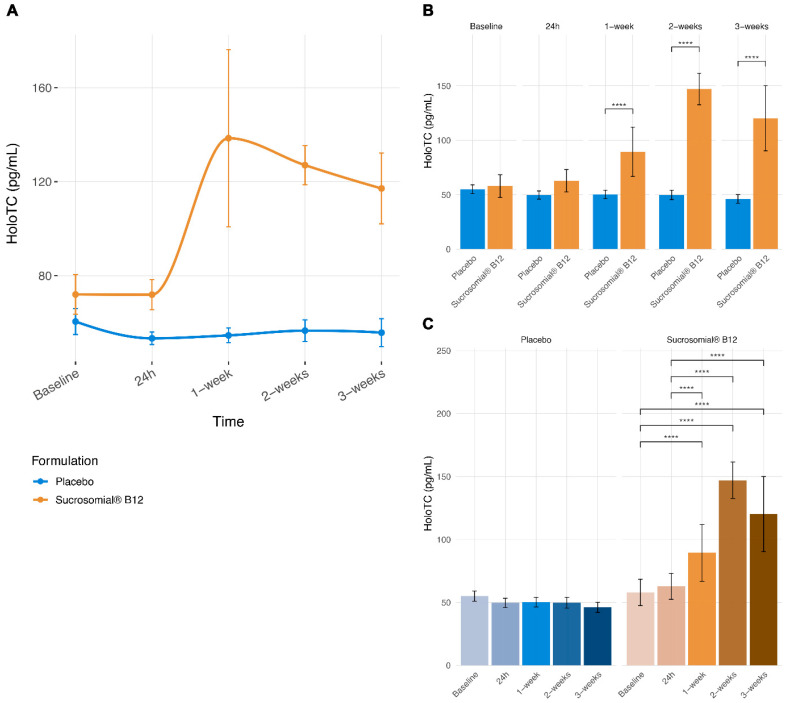
Longitudinal and comparative analyses of serum holotranscobalamin concentrations. (**A**) Connected scatterplots showing mean circulatory holotranscobalamin (HoloTC) concentrations (±SEM) over time in the Sucrosomial^®^ Vitamin B12 and placebo groups. Lines illustrate group-level temporal trends. (**B**) Barplots showing between-group differences in circulatory HoloTC concentrations at each timepoint. (**C**) Barplots showing within-group differences across timepoints for each formulation. Statistical comparisons were performed using LMM, and *p*-values were adjusted for multiple testing using the FDR method. At follow-up visits, values reflect only participants who had not yet reached the predefined clinical threshold (serum vitamin B12 ≥ 700 pg/mL) at earlier timepoints and therefore continued supplementation, as per study protocol. Significance levels are indicated as follows: *p* < 0.0001, ****. Numbers of participants contributing data at each timepoint were as follows: Placebo group Baseline *n* = 25, 24 h *n* = 25, 1-week *n* = 25, 2-week *n* = 25, 3-week *n* = 22; Sucrosomial^®^ vitamin B12 group Baseline *n* = 25, 24 h *n* = 25, 1-week *n* = 21, 2-week *n* = 10, 3-week *n* = 5.

Consistent with these findings, Post Hoc comparisons (Figure 4A,B) revealed no significant differences in circulatory HoloTC concentrations between groups at baseline or 24 hours after treatment initiation. In contrast, circulatory HoloTC concentrations were significantly higher in the Sucrosomial^®^ Vitamin B12 group compared with placebo from 1 week onward, corresponding to a 113% (ROC) at 1 week. This between-group difference remained significant at 2 weeks (ROC: 88%) and 3 weeks (ROC: 76%) (all FDR-adjusted *p* < 0.0001).

Within-group analyses (Figure 4A,C) showed no significant changes in circulatory HoloTC concentrations over time in the placebo group, with relative changes remaining below 12% across all follow-up visits and without evidence of a temporal trend. In contrast, participants receiving Sucrosomial^®^ Vitamin B12 demonstrated a marked increase in circulatory HoloTC concentrations beginning at 1 week (+92% relative to baseline), followed by persistently elevated levels at 2 weeks (+76%) and 3 weeks (+63%) (all FDR-adjusted *p* < 0.0001). No significant differences were observed among later follow-up timepoints within the Sucrosomial^®^ group, indicating early attainment of elevated HoloTC concentrations followed by stabilization.

Consistent with these findings, descriptive estimates showed that Sucrosomial^®^ Vitamin B12 supplementation resulted in an approximately two-fold increase in circulatory HoloTC concentrations at 1 week, with persistently elevated levels at subsequent timepoints, whereas no meaningful changes were observed in the placebo group.

### 3.4. Safety and Tolerability

The intervention was well tolerated over the three-week treatment period. No serious adverse events were reported, and no participants discontinued the study due to adverse events. Mild gastrointestinal symptoms were reported by one participant in the Sucrosomial^®^ vitamin B12 group and three participants in the placebo group. These events were transient, self-resolving, and did not require medical intervention. No clinically relevant abnormalities in vital signs were observed during the study. Treatment compliance was high, with a mean adherence rate exceeding 94% across both study groups.

## 4. Discussion

In this multicentre, double-blind, placebo-controlled randomized clinical trial, supplementation with a Sucrosomial^®^ vitamin B12 administered at a daily dose of 1000 µg for three weeks resulted in a rapid and consistent biochemical correction of vitamin B12 deficiency in metformin-treated adults with T2DM. The intervention produced clear and early separation from placebo, demonstrating that meaningful circulatory vitamin B12 repletion can be achieved within a short treatment window in a clinically challenging population characterized by pharmacologically impaired intestinal absorption. The present results align with prior randomized clinical evidence in healthy vitamin B12–deficient adults, in which an oral Sucrosomial^®^ vitamin B12 formulation was associated with faster and more consistent increases in circulating vitamin B12 concentrations compared with conventional supplements [32]. Within the broader framework of smart oral delivery systems, these findings highlight the translational potential of, food-compatible carrier platforms to improve the intestinal absorption of natural bioactive micronutrients in clinical nutrition.

Importantly, the observed increases were not limited to total circulating vitamin B12 but were also reflected in parallel improvements in the biologically active fraction, HoloTC. This finding supports the clinical relevance of the biochemical response and indicates that the observed increases translated into enhanced availability of vitamin B12 for tissue uptake, rather than representing isolated changes in circulating storage pools.

Beyond changes in group means, the study demonstrated a markedly higher likelihood and faster achievement of vitamin B12 normalization in participants receiving Sucrosomial^®^ vitamin B12 compared with placebo. Importantly, these treatment effects remained robust after adjustment for age, sex, BMI, and duration of T2DM, indicating that the observed differences were driven primarily by the intervention rather than baseline demographic or clinical imbalances. Notably, participants in the Sucrosomial^®^ group were older, had longer-standing T2DM, and a higher prevalence of diabetic neuropathy—factors typically associated with more severe malabsorption—yet a clear and rapid biochemical response was still observed, supporting the robustness of the delivery strategy under clinically more challenging conditions. The high proportion of individuals reaching adequate/upper-normal laboratory reference range circulatory B12 concentrations over a short period underscores the consistency of the response and highlights the potential utility of this strategy for the timely correction of deficiency in clinical practice.

Taken together, these findings show that an optimized oral Sucrosomial^®^ delivery strategy can overcome metformin-associated impairment of vitamin B12 absorption and achieve rapid, reliable restoration of both total and active vitamin B12 levels under rigorously controlled conditions, while maintaining a favourable safety and tolerability profile.

Metformin remains the cornerstone of T2DM management worldwide [19,20], and metformin-associated vitamin B12 deficiency is increasingly recognized as a clinically relevant and underdiagnosed complication [11,12,13,14,15,16,17]. Delayed identification and correction of vitamin B12 deficiency may contribute to hematological abnormalities and neurological symptoms, including peripheral neuropathy, which can overlap with or exacerbate diabetic neuropathy and complicate clinical assessment [13,23,27,28].

From a clinical perspective, an oral supplementation strategy that achieves rapid biochemical correction, is well tolerated, and is simple to administer has clear advantages over prolonged high-dose regimens or non-oral delivery routes. The ability to restore circulating vitamin B12 levels within a short timeframe may allow for earlier correction of deficiency, improved monitoring, and more individualized supplementation strategies, particularly in primary care and outpatient settings. Although the present study was not designed to evaluate dose-sparing, the rapid normalization observed with this delivery system raises the possibility that lower doses could be explored in future studies, provided that clinical efficacy and consistency of response are maintained.

From a safety perspective, Sucrosomial^®^ vitamin B12 supplementation was well tolerated over the three-week intervention period, with no serious adverse events or treatment discontinuations observed. The low frequency of mild, self-limiting gastrointestinal symptoms and the high level of treatment adherence are consistent with the favourable tolerability profile reported in previous vitamin B12 supplementation studies [32]. These findings support the suitability of this oral deliverystrategy for short-term correction of vitamin B12 deficiency in routine clinical practice.

**Figure 5:**
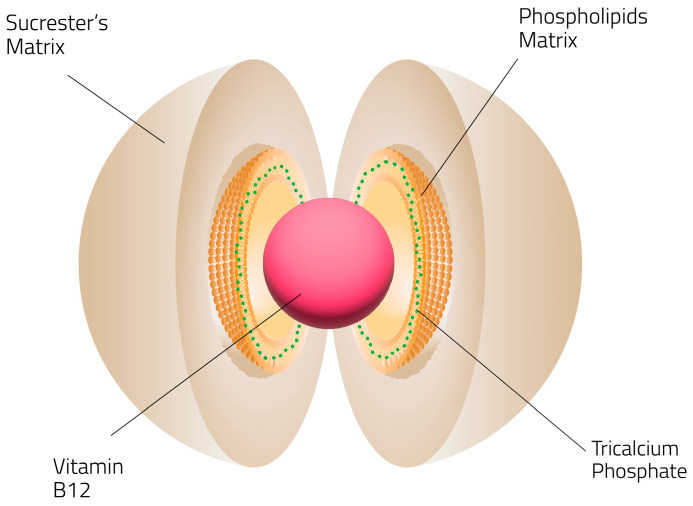
Schematic representation of the Sucrosomial^®^ vitamin B12. Vitamin B12 is protected by a phospholipids plus a sucrester’s matrix, designed to protect the nutrient during gastrointestinal transit and support intestinal uptake.

While the precise intestinal absorption pathways of Sucrosomial^®^ vitamin B12 in humans have not been fully elucidated, the Sucrosomial^®^ delivery system has demonstrated enhanced systemic exposure for multiple micronutrients across preclinical and clinical settings [33,34,35,36,37]. The ability of the sucrosome structure to protect nutrients during gastrointestinal transit and to interact with the intestinal epithelium via non-conventional transport mechanisms provides a plausible biological basis for the observed clinical effects (Figure 5).

Importantly, the present findings do not rely on assumptions regarding IF–dependent absorption and instead demonstrate functional efficacy in a population with known pharmacological impairment of this pathway. Further mechanistic studies may help clarify the specific transport processes involved, but such elucidation is not required to support the clinical relevance of the observed outcomes.

Previous studies in metformin-treated individuals with T2DM have established that vitamin B12 deficiency is correctable; however, they have largely relied on prolonged supplementation with conventional oral formulations or on alternative delivery routes that bypass gastrointestinal absorption altogether [27,39,40,41,42,43,44]. As a result, existing evidence provides limited insight into whether impaired intestinal absorption associated with metformin therapy can be effectively addressed through an optimized oral strategy, particularly within short treatment windows relevant to clinical practice. Moreover, most prior trials were open-label, heterogeneous in design, or focused on long-term outcomes rather than early biochemical correction. Moreover, they rarely evaluated the consistency or speed of normalization at the individual patient level. In contrast, the present multicentre, double-blind, placebo-controlled trial demonstrates that an optimized Sucrosomial^®^ delivery system can achieve rapid, consistent, and clinically meaningful restoration of circulating and active vitamin B12 levels using an oral approach, even in the setting of pharmacologically impaired absorption. By directly addressing limitations identified in earlier studies—namely, the lack of robust placebo-controlled data on effective oral supplementation—this study provides new evidence supporting the feasibility of rapid oral vitamin B12 repletion in metformin-treated T2DM patients.

Several limitations of the present study warrant consideration. The intervention period was intentionally short and focused on biochemical correction; as such, longer-term clinical outcomes, including neurological or hematological endpoints, were not evaluated. Although both total circulating vitamin B12 and the biologically active fraction, HoloTC, were assessed, downstream functional biomarkers of vitamin B12 status, such as homocysteine or methylmalonic acid, were not measured. Finally, mechanistic absorption pathways were not directly investigated, and the results should therefore be interpreted on the basis of demonstrated clinical efficacy rather than inferred mechanistic explanations.

Strengths of this study include its multicentre, randomized, double-blind, placebo-controlled design, which minimizes bias and enhances the internal validity of the findings. The study focused on a clinically well-defined population of adults with T2DM receiving metformin therapy, directly addressing a common and clinically relevant cause of vitamin B12 deficiency. The evaluation of both total circulating vitamin B12 and HoloTC allowed for a more comprehensive assessment of biochemical repletion. In addition, the emphasis on early response kinetics and the proportion of participants achieving normalization within a short treatment period provides clinically actionable information that is highly relevant to real-world practice. Finally, the favourable safety and tolerability profile observed under controlled conditions supports the feasibility of this oral supplementation strategy for routine clinical use.

## 5. Conclusions

In conclusion, this multicentre, double-blind, placebo-controlled randomized clinical trial demonstrates that a Sucrosomial vitamin B12 formulation can rapidly, reliably, and safely restore circulating vitamin B12 levels in adults with T2DM receiving metformin therapy. The ability to achieve consistent biochemical repletion, including increases in the biologically active fraction of vitamin B12, within a short treatment period highlights the effectiveness of this oral delivery strategy in a population with pharmacologically impaired vitamin B12 absorption. By addressing key limitations of previous studies and providing robust evidence for early correction using an oral approach, these findings support the clinical utility of Sucrosomial^®^ vitamin B12 in the management of metformin-associated vitamin B12 deficiency. Further studies are warranted to evaluate longer-term clinical outcomes and to inform evidence-based supplementation strategies in routine diabetes care.

## Figures and Tables

**Table 1:** Baseline demographic and clinical characteristics of the study population.

Category	Variable	Placebo (*n* = 25)	Sucrosomial^®^ B12 (*n* = 25)	*p*-Value
**Demographics**				
	Age (years)	43.7 ± 1.5	51.3 ± 2.2	0.007
	Sex (female/male)	12/13	10/15	ns
	Body temperature (normal)	25	25	ns
**Anthropometrics**				—
	Weight (kg)	76.6 ± 2.8	72.4 ± 2.6	ns
	Height (cm)	168.8 ± 1.6	171.4 ± 1.3	ns
	Body mass index (kg/m^2^)	27.8 ± 1.1	25.6 ± 0.9	ns
**Vital signs**				—
	Systolic blood pressure (mmHg)	124.6 ± 2.9	127.1 ± 3.2	ns
	Diastolic blood pressure (mmHg)	73.6 ± 2.4	77.6 ± 2.3	ns
	Heart rate (beats/min)	76.8 ± 1.5	78.3 ± 1.5	ns
	Respiratory rate (breaths/min)	16.9 ± 0.4	16.8 ± 0.4	ns
**Diabetes-related characteristics**				—
	Duration of T2DM (years)	5.5 ± 0.6	8.9 ± 0.9	0.003
	Insulin therapy (yes/no)	0/25	0/25	ns
	Diabetic neuropathy (yes/no)	20/5	12/13	0.038
	Diabetic retinopathy (yes/no)	10/15	7/18	ns
	Family history of T2DM (yes/no)	13/12	16/9	ns
**Lifestyle factors**				—
	Physical activity level	0.7 ± 0	0.8 ± 0	ns
	Diet type (C/O/V)	3/15/7	0/19/6	ns
	Smoking status (current/former/never)	3/1/21	4/3/18	ns
**Medical history**				—
	Gastrointestinal disorders (yes/no)	15/10	17/8	ns
	Surgical history (yes/no)	2/23	6/19	ns
**Supplement history**				—
	Previous B12 supplementation (yes/ no)	1/24	0/25	ns

Values are presented as mean ± SEM for continuous variables and as counts for categorical variables. Between-group comparisons for categorical variables were performed using Fisher’s exact test. For continuous variables, normality was assessed using the Shapiro–Wilk test within each study group, and between-group comparisons were conducted using either the Wilcoxon rank-sum test or the independent samples *t*-test, as appropriate. *p*-values are reported for descriptive purposes only; “ns” indicates not statistically significant (*p* ≥ 0.05). Abbreviations: T2DM, Type 2 Diabetes Mellitus; diet type abbreviations: C, carnivorous; O, omnivorous; V, vegetarian.

## Data Availability

The original contributions presented in this study are included in this article. Further inquiries can be directed to the corresponding authors.
